# DEVELOPMENT OF SELF-ASSESSMENT TOOLS FOR ORGANIZATIONS: PREVENTION AND DELAY OF DISABILITY PROGRAMS IN TAIWAN

**DOI:** 10.1093/geroni/igad104.2894

**Published:** 2023-12-21

## Abstract

This study aimed to develop a tool for service organizations to self-assess their characteristics and abilities of providing better disability prevention and function maintenance services. The development of the tool involved two stages. In the first stage, a self-assessment tool with 40 items was developed through literature based on Dunn’s policy evaluation framework. The tool was then subjected to two rounds of Delphi method, and pilot test. Six experts were invited, and the Delphi method results showed high consensus. Pilot test invited 170 service organizations to complete the survey and the results showed good internal consistency and validity with 20 items and six dimensions, including internal efficacy, responsiveness, professional, community collaboration, environmental factors, and sustainability (χ2[df=171, N = 170] = 282.88; p < 0.001; CFI = 0.923; RMSEA = .071; SRMR=.055). The sustainability (mean:4.20, SD: 0.61) and responsiveness (mean:4.20, SD: 0.59) showed the highest self-assess ratings. Combining internal resources and efficacy as internal efficacy showed good validity. and together with a new domain “internal efficacy” showed strong association with other domains. This tool could provide the program agencies to self-assess their ability to provide better disability prevention and function maintenance services. There were two new concepts, internal efficacy and sustainability, identified through the process which may indicate both of these two concepts are important to organization that provide health promotion services and culture-sensitive in Taiwanese society. Promoting these two concepts for future organizations is recommended.

